# Anaphylaxis to drug excipients

**DOI:** 10.1007/s40629-022-00214-9

**Published:** 2022-05-31

**Authors:** Wolfgang Pfützner

**Affiliations:** Allergy Center Hessen, Department of Dermatology and Allergology, University Hospital Marburg, Philipps-Universität Marburg, Baldinger Straße, 35043 Marburg, Germany

**Keywords:** Allergy, Polyethylene glycol, Polysorbate, Carboxymethyl cellulose, Patent blue, Gelatin

## Abstract

In addition to the therapeutic agent, drugs contain excipients such as stabilizers, preservatives, solubilizers, or dyes, some of which are identical to additives in foods. Anaphylaxis to these excipients is probably an underestimated problem. After the first descriptions of anaphylactic reactions to drug excipients appeared more than 30 years ago, the number of corresponding reports has increased significantly over the years. However, a diagnostic gap exists in the clarification of drug allergic reactions when the index product is not known and/or is not available for testing. In the present work, individual excipients are presented as examples for which publications on anaphylaxis are available. Furthermore, the options of allergological testing both in vivo and in vitro are discussed. The pathogenesis of such reactions is still unresolved in many cases, and current concepts are briefly presented in the conclusion. With increasing knowledge about anaphylaxis to drug excipients, it is assumed that these can then be recognized more often and diagnostically clarified.

## Background

Drugs are also referred to as medicines. The Latin word “medicare” means, among other things, “to cure” and “to treat,” but also “to make effective” or “to mix”. The latter illustrates that a drug usually does not contain only one substance (namely the active ingredient), but is composed of a mixture of different components. In addition to the therapeutic agent, these are excipients that fulfill different functions. For example, adjuvants are intended to enhance the desired therapeutic effect, stabilizers, preservatives and antioxidants to ensure a longer shelf life, and colorants and flavorings to favorably influence the qualitative properties of a drug. Many of these excipients are also found in foodstuffs, where they are given E numbers, which follow a general numerical classification system developed by the European Economic Community 50 years ago [1].

As important as excipients are for the manufacturing process, the stability and the therapeutic pharmacokinetics of drugs, their relevance for triggering undesirable adverse events, especially with regard to the development of allergic reactions, was considered to be very low until recently. However, numerous casuistic observations show that they are also capable of inducing anaphylaxis and should therefore be considered as potential elicitors in the diagnostic work-up, although the respective pathophysiological mechanism is not always clear. In the following, some examples of excipient-induced anaphylaxis by drugs are presented.

## Patent blue V (E 131)

Patent blue V is a synthetic triarylmethane dye used primarily in the food industry, so that sensitization is also possible via food intake [2]. In pharmaceuticals, it may be added to laxatives or cold remedies, for example, or used as a pure substance to mark sentinel lymph nodes or varicoceles. The incidence of intraoperative hypersensitivity to this dye is estimated to be up to 5% [3], which usually manifests as urticaria [4–6]. Allergological testing is primarily performed by means of skin tests [7], with the possible risk of permanent pigmentation, particularly in the case of intradermal tests. Furthermore, basophil activation tests (BAT) have been described for diagnostic purposes [8].

## Polysorbate 80 (E 433)

Polysorbate 80 is a polyoxyethylated ester of oleic acid and sorbitol, which is also commercially available as Tween® 80. It is a surfactant used in drug manufacture due to its emulsifying properties with good solubilization for lipophilic substances in aqueous bases. Thus, this excipient is particularly found in injectables such as local anesthetics, glucocorticosteroids that can be applied intramuscularly, and various biologics (e.g., omalizumab, adalimumab, ustekinumab, erythropoietin, darbepoetin). Reports of anaphylaxis to polysorbate 80 repeatedly appear in the literature, also described as cross-reactive reactions to polyethylene glycol (PEG; see below) [9–12]. A prick test can be performed for diagnosis [13]. False-positive reactions are possible in the intradermal test, which can be seen in particular when applying volumes > 0.02 ml [14].

## Gelatin (E 441)

Gelatin is a hydrocolloid mixture of animal products whose main ingredient is bovine or porcine collagen. Due to its hygroscopic properties, it is used in drug manufacture, among other things as a thickening agent to increase the viscosity of liquid drugs (e.g., Gelafundin®, Braun Melsungen AG, Melsungen, Germany). Other uses include as a stabilizer in the encapsulation of tablets, for coating suppositories and implants, and as a hemostyptic [15–20]. Anaphylactic reactions are IgE-mediated to both gelatin [15, 16] and the sugar galactose-α‑1,3‑galactose (α-Gal) contained therein [17–19]. Diagnostics include skin tests with the original preparation (prick test, if negative and application possible intradermal test starting at a dilution of 10^−3^) and the detection of specific IgE antibodies against gelatin and/or α‑Gal in commercially available assays. BAT are also possible [20]. If these tests are negative, oral provocation tests can be performed, which may include challenge with beef or pork ± cofactors of summation anaphylaxis in addition to the original preparation [21].

## Carboxymethyl cellulose (E 466)

Carboxymethyl cellulose is a synthetic polymer in which individual hydroxyl groups are linked to a carboxymethyl group. It shows a high ability to bind water and serves as a solubilizer, stabilizer, binder and lubricant. In pharmaceuticals, carboxymethyl cellulose is used as a “tablet disintegrant”; it is also an ingredient in injection preparations (e.g., glucocorticosteroids, hormones, X‑ray contrast media), laxatives, tear substitutes/ophthalmics and in hydrocolloid wound dressings. Anaphylaxis to carboxymethyl cellulose has been described after intra-articular injection of for example glucocorticosteroids, oral administration of BaSO_4_ contrast media and topical application of eye drops [22–25]. Diagnostics include skin tests, although the prick test is often negative, so that an intradermal test (dilution 10^−2^/10^−1^) must then be considered. Furthermore, different cellular in vitro assays can be performed [25, 26], and oral provocation tests are possible. While in various reports, however, no reaction was seen even with amounts as high as 250 mg, one patient developed urticaria after ingestion of 30 mg of carboxymethyl cellulose [26].

## Benzyl alcohol (E 1519)

Benzyl alcohol is a fragrance and aroma substance that can be found, for example, in oils of jasmine flowers, rosemary, cloves, meadow clover or balsam of Peru. It is used in the manufacture of pharmaceuticals as a solubilizer, preservative or antimicrobial substance. Examples of anaphylactic reactions include systemic hypersensitivity to the administration of chemotherapeutic agents (cytarabine, vincristine) and injection of heparin or cobalamin, respectively, to which benzyl alcohol was added [27, 28]. A positive immediate-type reaction was seen by intradermal testing at a concentration of 0.009% (control test negative) [28].

## Polyethylene glycol (E 1521)

Polyethylene glycol (PEG), also known as macrogol, is an ethylene oxide polymer whose molecular weight is between 200 and 35,000 g/mol, depending on the number of ethylene oxide molecules. It is highly hygroscopic and thus exhibits very good water solubility. In the pharmaceutical industry, it is used as a solubilizer in which drugs or their surrounding shells (e.g., nanoparticles) are pegylated, furthermore as a “disintegrant” of tablets or as a pure substance serving as a laxative. Anaphylaxis to PEG has been described not only in individual case reports but also in case series [9, 29, 31]. Typical triggers are laxatives (> 50%), injection solutions (e.g., glucocorticosteroids), non-steroidal anti-inflammatory drugs and antibiotics [29]. Diagnostic work-up includes skin prick and intradermal testing (max. concentration 10^−1^), for which PEG of higher molecular weights (≥ 2000) should be used. Furthermore, BAT (both with PEG as pure substance and pegylated phospholipid particles, commercially available; Table 1) and oral provocation tests (e.g., with macrogol) can be performed [9, 29–31].

**Table 1:** Diagnostics of anaphylaxis to drug excipients

Excipient	Diagnostics		
	Skin tests	Laboratory tests	Challenge tests
Patent blue V	Prick (10^−1^/pure) [6] i. d. (10^−2^) [6]	BAT^a^ [7]	–
Polysorbate 80	Prick (pure) i. d. (10^−2^, 10^−1^) [9]	BAT^a^	200 mg^b^ p.o. [9]
Gelatin (Galactose-α-[1,3]-galactose)	Prick (10^−1^, pure) [16] i. d. (10^−3^–10^−1^) [16]	IgE^c^ [14, 16] BAT^a^ [19]	Beef-/porcine meet (α-Gal) [20]
Carboxymethyl cellulose	Prick (10 µg/ml) [24] i. d. (10^−2^–10^−1^) [24]	CAST^a^ [24] BAT^a^ [25]	30 mg^b^ [25]
Benzyl alcohol	i. d. (0.009%) [27]	–	–
Polyethylene glycol	Prick (10^−2^, pure) [28, 32] i. d. (10^−3^–10^−1^) [28, 32]	IgE^a^ [8] BAT^d^ [32]	7.1 g^e^

*BAT* Basophile Activation Test, *CAST* Cellular Allergy Stimulation Test, *i.* *d.* intradermal, *p.o.* per os

^a^No commercial test

^b^Maximal dose

^c^Thermo Fisher, Freiburg, Germany

^d^Bühlmann, Basel, Switzerland

^e^Maximal cumulative dose

## Lactose

Lactose is a disaccharide of the sugars galactose and glucose and is added as a stabilizer to drugs such as dry powder inhalers, glucocorticosteroid injection solutions or vaccines. Anecdotal reports of anaphylaxis to lactose in drugs in patients with cow milk allergy attribute these to impurities with milk protein components or galactose-containing oligosaccharides similar to galactose-α‑1,3‑galactose, rather than allergic reactions to lactose itself [32, 33].

## Pathogenesis and conclusion

The pathogenesis of anaphylaxis to excipients in drugs is often unclear. Positive prick tests and BAT suggest IgE-mediated immediate reactions in at least some of the reactions [6–9, 13, 26, 28–30]. For gelatin- and α‑Gal-mediated reactions, IgE-mediated sensitizations are well documented, and detection of PEG-specific IgE antibodies has also been reported [9]. It is speculated whether some excipients become reactive antigen only after binding as a hapten to a carrier [34]. Another possibility discussed is a complement-mediated immune response, as in the case of PEG-induced reactions [29]. The observation that intramuscular injection of a pegylated nanoparticle COVID-19 (coronavirus disease 2019) vaccine was easily tolerated by a patient who had a history of anaphylactic reactions after ingestion of PEG-containing drugs and showed positive skin tests or BAT to PEG or PEG-containing vaccines, respectively, may suggest that the form of administration, the mode of presentation, or the composition of an excipient could be significant in triggering anaphylaxis [35]. More research is needed in this area to develop a better understanding of the pathogenesis and clinical relevance of anaphylactic reactions to drug excipients.

## Abbreviations

α‑Gal: Galactose-α‑1,3‑galactose
BAT: Basophil activation tests
PEG: Polyethylene glycol
